# High Prevalence but Insufficient Treatment of Iron-Deficiency Anemia in Patients with Inflammatory Bowel Disease: Results of a Population-Based Cohort

**DOI:** 10.1155/2012/595970

**Published:** 2012-07-30

**Authors:** Claudia Ott, Anne Liebold, Angela Takses, Ulrike G. Strauch, Florian Obermeier

**Affiliations:** Department of Internal Medicine I, University of Regensburg, 93042 Regensburg, Germany

## Abstract

*Background*. Iron-deficiency anemia is described to be a common problem in patients with inflammatory bowel disease (IBD), which is frequently associated with a reduced quality of life. Therefore, the aim of this study is to assess the prevalence of iron deficiency anemia in a population-based cohort at time of first diagnosis and during the early course of the disease. *Methods*. As far as available, lab values of patients registered in the population-based “Oberpfalz cohort” were screened. In anemic patients, we further investigated all laboratory results to differentiate between iron deficiency and other reasons for anemia. All patients with any kind of anemia were interviewed separately according to symptoms of iron-deficiency anemia and administration of iron. *Results*. In total, we evaluated hemoglobin values of 279 patients (183 Crohn's disease, 90 ulcerative colitis, and 6 indeterminate colitis). Lab data which allowed further differentiation of the type of anemia were available in 70% of anemic patients, in 34.4% values of iron, ferritin and transferrin saturation had been measured. At time of first diagnosis, an iron-deficiency anemia was diagnosed in 26 of 68 patients with anemia (38.2%, 20 CD, 4 UC, and 2 IC patients), but only 9 patients (34.6%) received subsequent iron therapy. After one year, 27 patients were identified to have an iron-deficiency anemia (19 CD, 8 UC), 20 of them were treated with iron (71.4%). Of 9 patients with proven iron-deficiency anemia at time of first diagnosis and subsequent administration of iron, 5 (55.5%) had iron-deficiency anemia despite permanent treatment after one year. In total, 38 patients (54.3%) did not receive any iron substitution at all despite of proven iron-deficiency anemia, and only 13 patients of 74 patients were treated with intravenous iron (17.6%). *Conclusion*. We found a high prevalence of iron-deficiency anemia at different points during the early course of disease in this population-based cohort of IBD patients. Surprisingly, only in one-third of patients with proven anemia, further diagnostic approach was undertaken. Even patients with diagnosed iron-deficiency anemia were infrequently and inconsequently treated with iron preparations, despite the high impact on quality of life.

## 1. Background

The main entities of inflammatory bowel diseases (IBDs) are Crohn's disease (CD) and ulcerative colitis (UC), which are chronic diseases of multifactorial pathogenesis with increased susceptibility caused by genetic factors which might be influenced by immunological mechanisms and environmental factors [1]. The main symptoms of CD and UC are abdominal pain and (bloody) diarrhea, partly combined with other complications as extraintestinal manifestations affecting joints, skin, or other organ systems. Besides intestinal symptoms, anemia is one frequently occurring problem in patients with IBD with significant impact on the quality of life in affected patients [2, 3]. 

Iron-deficiency anemia (IDA) is the most common reason for anemia in patients with IBD, mainly caused by chronic blood loss due to mucosal damage on the one hand and reduced iron absorption in inflamed mucosa in the duodenum and upper jejunum in CD patients on the other. Also dietary intake may influence iron deficiency as patients with IBD are reported to avoid food which may increase abdominal symptoms [4]. 

The second major cause for anemia in IBD is the anemia of chronic disease (ACD), which is associated with chronic activation of cell-mediated immunity. But also decreased absorption of vitamin B12 and folate in inflamed regions may result in anemia. In addition, different therapeutical agents for IBD causing myelosuppression (such as azathioprine and 6-mercaptopurine) can induce anemia [5]. Anemia in patients with IBD is mostly a combination of different mechanisms, mainly iron-deficiency anemia and anemia of chronic disease [5].

Depending on study design, the patient population considered, and definition of anemia, 6–74% of patients with IBD suffer from anemia [6]. The prevalence of iron deficiency in patients with IBD even varies between 36 and 90% [7]. As iron-deficiency anemia may reduce the quality of life and also the ability to work [2, 3], it seems to be reasonable that in IBD patients with anemia, not only a disease-specific treatment has to be administered, but also an existing iron deficiency should be recognized and treated if necessary to improve patients' quality of life. 

As most studies on the prevalence of iron-deficiency anemia in patient with IBD are retrospective or are surveys from referral centers, the aim of this study was to assess the prevalence of iron-deficiency anemia in a population-based cohort at time of first diagnosis and during the early course of the disease. In addition, we evaluated the frequency of treatment, the way of administration, and effectiveness of subsequent medical therapies in this population-based setting.

## 2. Material and Methods

### 2.1. Population

The population investigated in this study is described elsewhere [8]. In summary, we raised a prospective population-based cohort in the rural region of Oberpfalz (Bavaria, Germany) setting up a network of reporting clinicians and general practitioners including internists, gastroenterologists, surgeons, and paediatricians, in hospitals as well as in private practice. This study was approved by the institutional ethics committee on human studies according to criteria of the modified Helsinki Declaration of 1983.

Recruitment for this study started at January 01, 2004. Data up to January 31, 2009 are reported. For each patient, a standardized data form was completed at the time of first diagnosis by the attending physician including demographic data (date of birth, gender, and place of residence), onset of symptoms, date of diagnosis, extent of disease, familial occurrence of IBD, extraintestinal manifestations, and actual laboratory tests. After informed consent, all patients with proven anemia were contacted by the study centre personnel. The interview included questions on anemic symptoms, kind of substitution (oral versus parenteral), digestibility of the medication, duration of treatment, disease-specific treatment, and special dietary habits (especially vegetarian). If the patient was not able to give details on the medical treatment, the attending physician was contacted after special informed consent. 

All laboratory results were collected at three observation points: at time of first diagnosis, one year after first diagnosis, and at the end of observation in January 2009. Laboratory investigations of interest included hemoglobin (hb), iron, ferritin, transferrin, soluble transferrin receptor (sTfR), transferrin saturation, c-reactive protein (CRP), and serum levels of folate and vitamin B12. As this study is population based and not all patients have been investigated in our referral center, not in all included patients a total body iron status and serum vitamin levels have been conducted. In those patients, an iron-deficiency anemia was presumed in patients with microcytosis and normal CRP if iron, ferritin, and transferrin saturation were not available. In addition, not all patients gave informed consent to contact them, and few colleagues reported patients on an anonymous basis; therefore, in some patients, the followup could not be evaluated at every observation point.

As the predominance of laboratory investigations were performed at one main institute, the presence of anemia was defined according to the ranges of this lab with hemoglobin levels lower than 13.4 g/dL for men and 11.8 g/dL for women. All reference values are given in Table 1.

### 2.2. Statistics

Statistical analysis was performed using SPSS software (SPSS for Windows 17.0, Chicago, IL, USA). Data are given as numbers and percentages, medians, and range. 

Exploratory analysis was performed using chi-squared testing (or *t*-tests for continuous variables) based on a 95% confidence level (two sided).

## 3. Results

During the study period between January 1, 2004 and January 31, 2009, 456 patients with newly diagnosed IBD were screened for anemia. Hemoglobin values were available in 279 patients (183 CD, 90 UC, and 6 IC) during the study period. In 90 of the 279 patients (32.3%) with documented hb levels, anemia was present at any point during the study. In 68 patients (75.5% of all anemic patients), anemia was found at time of first diagnosis, and 22 patients developed anemia not later than one year after first diagnosis. There was no statistically significant difference between affected patients in relation to gender or disease entities (see Table 2).

**Figure 1:**
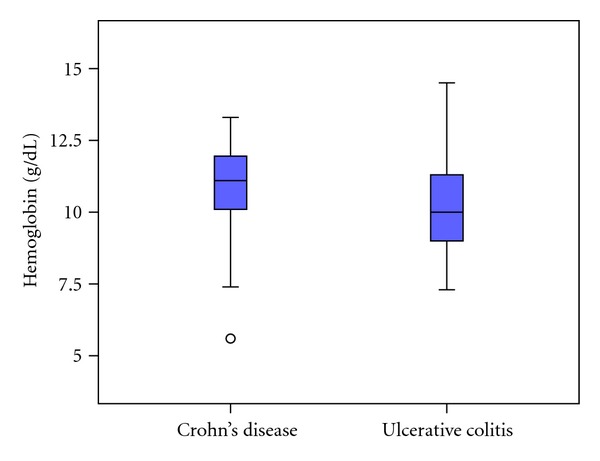
Hb-levels at first diagnosis.

Median age of male anemic patients with CD was 21 years (7–57 years), and female patients was 22.5 years (5–71). In UC patients, males were 21.5 years (6–74 years), and female anemic patients were slightly older with 38 years (12–74 years). Median hb values in CD patients with anemia were 11.1 g/dL (5.6–13.3 g/dL); in UC patients, severe anemia was seen more frequently, and the median hb value was 10.0 g/dL (7.3–14.5 g/dL) (Figure 1).

At the final observation point, the median hb levels in UC patients slightly increased to 11.3 g/dL (9.4–16.6 g/dL, *P* = 0.001), but did not increase in CD patients with median hb levels of 10.0 (8.8–15.0 g/dL, *P* = 0.9).

## 4. Prevalence of Iron-Deficiency Anemia 

63 (70%) patients of all 90 patients with anemia met the criteria of iron-deficiency anemia. In 31 (34%) of all patients with anemia, values of iron, ferritin, and transferrin saturation had been measured; in another 32 patients, an iron-deficiency anemia was diagnosed due to microcytosis and CRP levels. At time of first diagnosis, an iron-deficiency anemia was diagnosed in 26 of 68 patients with anemia (38.2%, 20 CD, 4 UC, and 2 IC patients). 

One year after first diagnosis of IBD, 27 patients with proven anemia were diagnosed having an iron-deficiency anemia (61.3%). At that point, mainly CD patients were affected by iron-deficiency anemia (19 CD versus 8 UC patients). 

At the last observation point in January 2009, 17 of 19 patients with proven anemia were diagnosed as having an iron-deficiency anemia (89.5%). 

**Figure 2:**
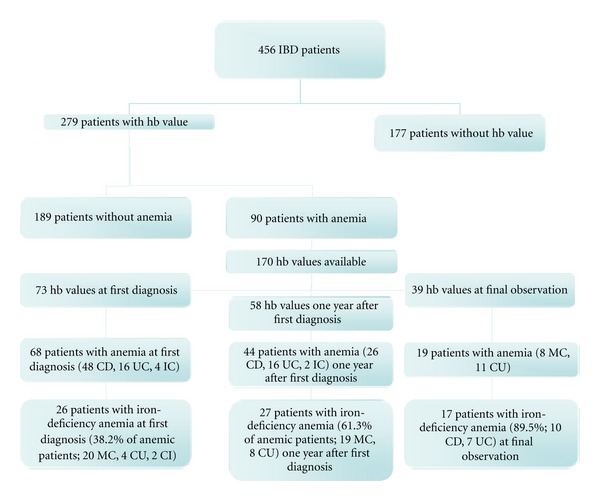
Characteristics of all patients with anemia during the different observation points.

A detailed overview of all patients with anemia is given in Figure 2.

Details concerning the disease activity were available in 42 patients (60%) with proven iron-deficiency anemia. In total, 26 patients achieved full clinical remission (65%), whereas 10 patients had a chronic active course of their disease (23.8%). The remaining 6 patients (14.2%) reported intermittent flares during the observation period. 

## 5. Frequency and Type of Iron Substitution

In 23 of 26 patients with proven iron-deficiency anemia at time of first diagnosis, detailed information on medical treatment was available. Only 9 patients (35% of all patients with iron-deficiency anemia at that point) were substituted with iron. Six of the 9 patients treated with iron were seen at a tertiary referral centre (66%). All patients were treated with oral iron formulations. 

After one year, in 25 of 27 patients with iron-deficiency anemia, further information of treatment was obtained. In total, 18 of 25 patients (72%, 12 CD, and 6 UC) received iron substitution at that point. Of 17 patients with iron-deficiency anemia seen at a referral centre, 14 were recommended to supplement iron (82.4%)—4 of 8 patients (50%) seen by their family doctors received iron. Fourteen patients were treated orally, 4 patients with intravenous iron. 

At the last observation point, only 5 of all 17 patients (29.4%) with iron-deficiency anemia were treated with oral iron substitution, and no patient was medicated with intravenous iron. All of the patients treated with iron were outpatients at a referral centre. 

Considering the entire study period, 32 patients of all 70 patients (46%) with proven iron-deficiency anemia at any point of observation were treated with iron substitution. Patients were mostly prescribed oral iron formulations (*n* = 27, 39%), and 5 patients were treated with intravenous iron (7%). In total, 38 patients (54.3%) did not receive any iron substitution at any point despite proven iron-deficiency anemia. 

## 6. Duration of Iron Substitution and ****Digestibility

46% (*n* = 27) of all patients with iron-deficiency anemia were treated with iron substitution. In 22 of all 27 patients with oral iron substitution, information of the prescribed iron formulation was available. Most patients were treated with oral iron sulphate formulations (*n* = 21), and one patient received iron gluconate (II). One additional patient reported to pay special attention on iron-rich nutrition.

**Figure 3:**
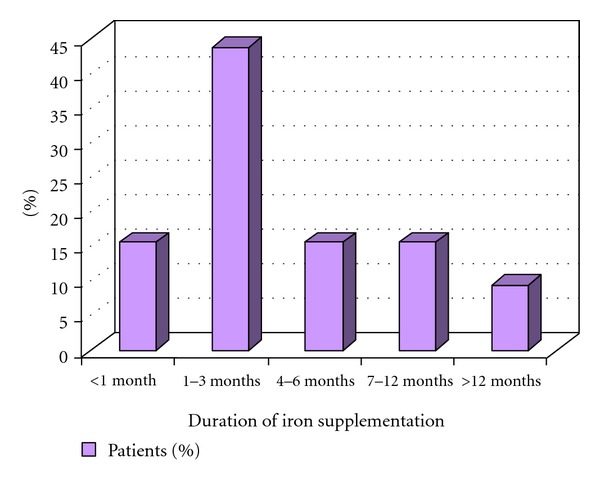
Duration of iron supplementation.

Patients most frequently received supplement iron over 1–3 months of time (Figure 3).

**Figure 4:**
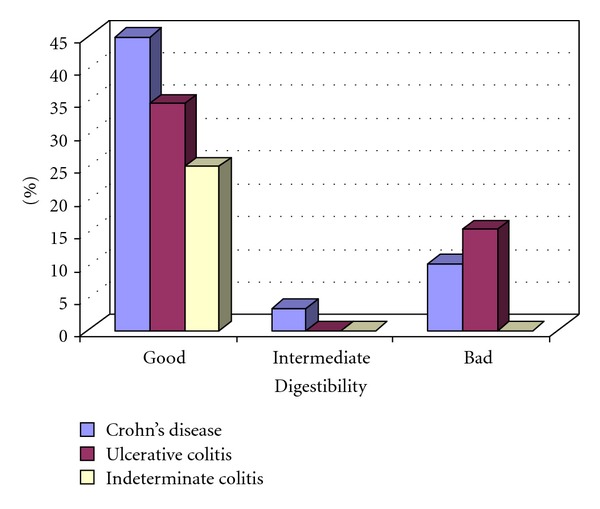
Digestibility of oral iron preparations.

Less than half of all patients treated with oral iron preparations (47.1%) assessed the digestibility of formulations as “good” (Figure 4). Most frequently reported side effects were constipation, nausea, and abdominal pain.

No patient receiving intravenous iron supplementation (*n* = 5) complained of any side effects of the specific therapy. An improvement of typical symptoms of anemia as fatigue and increased need of sleep were reported in 27.5% of patients. 

In 8 of the 32 (25%) treated patients with iron-deficiency anemia, the anemia persisted despite of iron supplementation during the observation period. Half of the patients with persisting anemia reported a chronic active course of their disease. Details of the patients with iron-deficiency anemia despite treatment are given in Table 3.

Seven of the 8 patients with persisting iron-deficiency anemia were seen at a tertiary referral centre. Of these 7 patients, 5 patients (71%) were recommended to switch to intravenous iron application. Subsequently, three patients received intravenous iron therapy at the next observation point.

## 7. Discussion

Anemia is a common problem in patients with IBD, especially in patients with CD due to malabsorption, malnutrition, resections causing iron deficiency, vitamin B12, and folic acid deficiency leading to a multifactorial pathogenesis of anemia [5, 9]. Several studies reported a markedly variable prevalence of anemia in IBD patients ranging between 6 and 74% [7, 10] with a mean prevalence of 17% [7]. 

As many reports have focused on the important problem of anemia in IBD patients [9, 11, 12], Gasche et al. finally developed the first simple guidelines for diagnosis and management of iron-deficiency anemia in IBD patients in order to improve patients care and quality of life [13]. These guidelines may be used as basic concept to develop country-specific recommendations as published most recently by the Austrian IBD working party [14].

Despite of the increasing attention to the problem of anemia in the predominantly young patients with IBD, our study of a population-based cohort showed a high prevalence of anemia at different points of the disease. At time of first diagnosis, anemia was found more frequently in our patients than at points of followup. At first diagnosis, anemia of chronic disease was predominant, whereas during followup, iron deficiency became the most relevant reason of anemia. These findings are in line with data of Bergamaschi et al. [6]. In his study, he also described a strong association between the severity of anemia and disease activity. Unfortunately, in our population-based setting, not all necessary information was available in every patient to define disease activity according to CDAI or Mayo scores, but on clinical assessment, no clear association with disease activity and anemia became obvious in our series. 65% of patients with proven anemia were reported to be in clinical remission. As shown in Table 3, half of patients with persisting iron-deficiency anemia despite of iron supplementation were in clinical remission. A possible explanation of these findings might be the population-based character of our series, as not only outpatients of a tertiary referral centre were included in this study, but also patients with less severe forms of IBD, which are mainly treated by their family doctors. In this context, reasons for the insufficient response to the treatment might be underdosing of iron supplementation, subclinical inflammation of the underlying disease, or lack of adherence of the patient. 

Due to the population-based design, another weakness of the study is the lack of the total iron body status in every patient. Therefore, we presumed an iron-deficiency anemia in patients with microcytosis and normal CRP if iron, ferritin, and transferrin saturation were not available according to the recommendations of the guidelines of Gasche et al. [14] with the limitation of factors influencing the MCV, especially the use of azathioprine, which can lead to increased MCV. In total, 19 anemic patients of our cohort were using azathioprine at any point during the observation period. Of those, only 2 (10.5%) patients were diagnosed not having an iron-deficiency anemia due to the criteria not having microcytosis and normal CRP. 

Another limitation of the study is the fact that hemoglobin levels were not available in all patients diagnosed with IBD as not all patients gave informed consent to contact them, and few colleagues reported patients on an anonymous basis. This possible bias could influence the prevalence of anemia resulting in overexpression of patients with active disease which were more likely to undergo intensive laboratory workup. 

In fact, treatment of iron-deficiency anemia and especially the duration of treatment seems to be inefficient in daily practice. In a recent published cross-sectional study from Switzerland, Voegtlin et al. reported 40% and 43% of patients being treated with supplements as iron, vitamin B12, and folic acid in private practice and university hospitals, respectively, [15]. Gisbert et al. showed oral and intravenous iron treatment to be safe and well tolerated in IBD patients with good clinical response in both formulations [16]. But several clinical trials described less tolerability of oral formulations with mainly gastrointestinal side effects and prolonged response to intravenous iron substitution [17, 18]. In addition, studies in animal models suspect oral iron formulations to increase disease activity in IBD and even the risk of development of colorectal cancer [7]. 

In their guidelines on the management of iron-deficiency anemia, Gasche et al. recommend an intravenous iron application, especially in patients with severe anemia [13]. However, these guidelines seem to be far from entering daily routine. In our population-based cohort, none of the patients was treated with intravenous iron at time of first diagnosis although one patient had a minimum hb level of 5.6 g/dL. As one possible reason for this reservation of administration of intravenous formulations, one could mention the potential risk of anaphylactic reactions described for low- and high-molecular-weight iron dextran [19]. But since several dextran-free formulations of intravenous iron are available as iron gluconate, iron sucrose, and ferric carboxy-maltose, such side effects were not observed up to now [14]. In addition, the novel formulations have shown a rapid increase in hb levels as well as in storage iron with a fast improvement of patients' quality of life [2, 20, 21]. 

And despite the knowledge that the quality of life in anemic IBD patients tends to be similar to patients with advanced cancer [22], more than half of patients with proven iron-deficiency anemia were not administered iron at all, which is an alarming result of our series.

To our knowledge, this is the first analysis investigating the care of anemic IBD patients in daily routine in a population-based cohort at first diagnosis and during the early course of their disease. The awareness on the impact of anemia in this cohort seems to be lower than expected leading to inconsequent diagnostic approach and even lack of treatment. Therefore, also non specific treatment beside, for example, immunosuppressive therapies should be intensified to improve the care of the patients especially with respect to the quality of life. 

## 8. Conclusion

This population-based series on the prevalence and the care of IBD patients with anemia showed a high prevalence of anemia at first diagnosis and during the early course of the disease. Results concerning the treatment of iron-deficiency anemia are alarming as only a minority of patients are treated effectively in daily routine although simple guidelines for diagnosis and management of iron-deficiency anemia in IBD patients exist. Therefore, one goal in the care of IBD patients is to intensify the awareness of the frequent codiagnosis of anemia leading to reasonable diagnostic approach and effective treatment to increase patients' quality of life.

## Figures and Tables

**Table 1:** Reference values of laboratory investigations.

Laboratory parameter (units)	male	female
hb (g/dL)	13.4–17	11.8–16
Ferritin (*μ*g/L)	15–400	18–120
Transferrin (g/L)	2–3.6	
sTfR (mg/dL)	0.83–1.76	
Transferrin saturation (%)	16–50	
White blood count (/*μ*L)	3.800–10.500	
Hematocrit (%)	40–52	37–48
MCV (fl)	85–98	
MCH (pg)	28–34	
CRP (mg/L)	<5	
Vit. B12 (pg/mL)	210–910	
Folate (ng/mL)	3–15	
Iron (*μ*g/dL)	50–160	50–150

**Table 2:** Gender-specific characteristics of the patients with anemia related to all patients with hb levels.

Gender	Type of IBD	
	Crohn's disease (% of all patients with hb levels)	Ulcerative colitis (% of all patients with hb levels)
Male	*n* = 28 (34.1%)	*n* = 14 (31.8%)
Female	*n* = 32 (30.7%)	*n* = 12 (26.1%)

**Table 3:** Details on the 8 patients with persisting iron-deficiency anemia despite of treatment.

Type of IBD	Hb level (g/dL)	Way of iron administration	Duration of substitution (months)	Disease-specific treatment	Activity of IBD
Crohn's disease	11.1	oral	>12	—	Remission
Crohn's disease	10.7	Oral and i.v.	1–3	Prednisolone	Chronic active
Crohn's disease	13.3 (transferrin saturation 9%)	oral	1–3	—	Remission
Crohn's disease	7.4	oral	1–3	Methotrexate	Chronic active
Ulcerative colitis	8.4	oral	>12	Mesalamine, prednisolone	Chronic active
Crohn's disease	9.7	i.v.	1–3	Methotrexate	Chronic active
Ulcerative colitis	9.4	oral	<1	Mesalamine, prednisolone	Remission
Ulcerative colitis	11.9	oral	1–3	Mesalamine	Remission
